# Visual perceptions of portion size normality and intended food consumption: A norm range model

**DOI:** 10.1016/j.foodqual.2018.10.003

**Published:** 2019-03

**Authors:** Ashleigh Haynes, Charlotte A. Hardman, Alexis D.J. Makin, Jason C.G. Halford, Susan A. Jebb, Eric Robinson

**Affiliations:** a Institute of Psychology, Health & Society, University of Liverpool, L69 7ZA, UK; b Nuffield Department of Primary Care Health Sciences, University of Oxford, UK

## Abstract

• Smaller portion sizes are associated with lower energy intake.
• We test a norm range model of the portion size effect on intended intake.
• A wide range of portion sizes were perceived as normal.
• Portions perceived as normal did not prompt intended compensatory eating.
• Portions perceived as smaller than normal prompted intended compensation.

## 1 Introduction

Portion size refers to the amount of food served that is available for immediate consumption in a single eating occasion (Almiron-Roig, Navas-Carretero, Emery, & Martínez, 2018). There is now substantial evidence that larger portions of food promote greater intake of that food (Hollands et al., 2015, Kelly et al., 2009, Zlatevska et al., 2014). Portion sizes of many foods have increased over the past four decades (Nielsen and Popkin, 2003, Smiciklas-Wright et al., 2003, Wrieden et al., 2008), which has coincided with a dramatic rise in obesity (Livingstone and Pourshahidi, 2014, Ng et al., 2014). Reducing the portion size of commercially available foods has therefore been highlighted as a promising strategy to reduce energy intake and tackle obesity (Marteau, Hollands, Shemilt, & Jebb, 2015).

An issue with reducing portion size is that if too large a reduction is made, consumers may compensate for the smaller size by eating more than one portion of that food or by consuming more of other foods. This may result in total energy intake that is equal to (compensation) or exceeds (‘overcompensation’) the amount that would have been consumed from a standard, non-reduced portion. For example, once the decision is made to consume a second serving or another food, due to there being a zone of ‘biological indifference’ for food intake (Herman & Polivy, 1983) consumers may be able to eat all of the additional serving and motivated to do so because of unit bias (the tendency to consume the entirety of a single entity rather than a fraction, e.g., one whole plateful or piece of food, Geier, Rozin, & Doros, 2006).

We are aware of three studies that have directly examined the effect of reducing portion sizes on energy intake. Rolls, Roe, and Meengs (2006b) demonstrated that energy intake across a two day period was 10–12% lower when participants were served meals that were reduced by 25% relative to a standard portion. Similarly, Lewis, Ahern, et al. (2015) found that energy intake in subsequent meals was not significantly different following a breakfast portion reduced by 40% vs. a standard portion. The magnitude of reduction to portion size in these studies did not result in participants fully compensating at later meals. In a 6-month RCT, participants who were provided with a lunchbox containing portions of food reduced by 50% ate significantly less at lunchtime than participants who were provided with ‘typical’ food portions (French et al., 2014). However, the reduced lunchtime portions did not significantly reduce daily energy intake or body weight, suggesting that participants compensated for the reduction by eating more of other foods. Understanding the point at which reductions to portion size result in compensatory eating is therefore crucial to inform effective reductions that reduce total energy intake.

One explanation for the effect of portion size on consumptions is that it provides a visual norm or a guide for how much is appropriate to eat. For example, Diliberti, Bordi, Conklin, Roe, and Rolls (2004) found that while increasing the portion size of an entrée by 50% increased energy intake relative to a standard portion by 43%, both standard and larger portions were perceived as equally as ‘appropriate’ by consumers. Similarly, Kerameas, Vartanian, Herman, and Polivy (2015) found that participants who were served a large portion of cookies reported that a larger portion was appropriate to eat and subsequently consumed more than those served a smaller portion. However, the relationship between increasing portion size and greater food intake begins to plateau at extremely large portion sizes (Hollands et al., 2015, Zlatevska et al., 2014). Parallel to this, while a range of portions are likely to be considered ‘normal’, it is unlikely that very small or very large portions are perceived as such (Herman & Polivy, 2005).

### 1.1 The norm range model and the present research

While visual perception of volume can be inaccurate in humans (Ordabayeva & Chandon, 2013), we theorise that whether or not a portion is visually perceived as being ‘normal’ in size determines how much of that portion a consumer intends to eat. This theory is in part based on work suggesting that altering how ‘normal’ in size a portion of food appears affects evaluations of intended consumption (Robinson et al., 2016) and that the amount of food consumers intend to eat relates to how much they subsequently do eat (Robinson, te Raa, & Hardman, 2015). Humans often rely on heuristics for efficiency when making perceptual judgments (Harnad, 1987). One example is categorical perception, whereby physical stimuli that vary continuously (e.g., by ‘attractiveness’) are perceived as belonging to one of a limited number of discrete perceptual categories, (e.g., ‘attractive’ vs. ‘unattractive’) (Calder et al., 1996, Harnad, 1987, Tovée et al., 2012). While there is evidence that some categories are partially innate (e.g., phoneme categories in speech perception), the location of boundaries between categories can also be generated or modified by learning (Harnad, 1987, Lawrence, 1950, Logan et al., 1991). The first proposition of the norm range model is that portion size (a stimulus that varies along a physical continuum) is categorically perceived as ‘normal’ or ‘not normal’ in size. We speculate that most consumers lack certainty about how much is an appropriate amount of food to consume, which is supported by the observation that people can be inaccurate at judging food volume and energy content, and perceive serving size guidelines as conflicting and confusing (Bucher et al., 2017, Nørnberg et al., 2014, Ordabayeva and Chandon, 2013, Ordabayeva and Chandon, 2016, Rozin et al., 1996; M Spence et al., 2013). As a result, we expect that most consumers will categorise a wide range of portion sizes as ‘normal’.

To test the norm range model, we conducted two virtual experiments. Our first aim was to identify the ‘norm range’ of portion sizes for several different foods, by examining the portion sizes for which a majority of people judged as being ‘normal’. Our second aim was to examine evidence for the categorical perception of portion size normality. A key feature of categorical perception is that stimuli that are from different perceptual categories (e.g., ‘normal’ and ‘not normal’) are more discriminable than stimuli from within the one perceptual category (e.g., two ‘normal’ stimuli) (Calder et al., 1996, Harnad, 1987). Relative judgments of stimulus features such as size and presentation interval have been used in previous studies investigating perceptual discrimination (Lapid et al., 2008, Lapid et al., 2009). To test whether portion size normality was perceived categorically, participants judged the relative size of two simultaneously displayed portions in Study 2. We predicted that the relative size of portion sizes that crossed the lower or upper norm range boundary (from different perceptual categories) would be discriminated more quickly and accurately than those that were located within an individual’s norm range (from the same perceptual category).

**Fig. 1:**
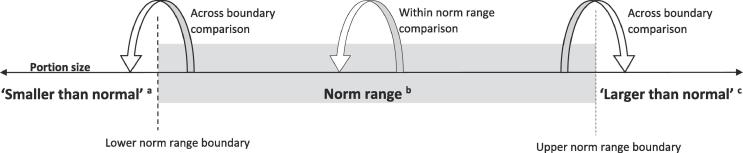
Norm range model. Hypothesised intended consumption: ^a^intended consumption of full portion served and more (compensatory eating), ^b^intended consumption of full portion served and no more, ^c^intended consumption of less than full portion served.

Our third aim was to test the proposition of the norm range model that if a food portion is perceived as being ‘normal’ in size, this is likely to be used as a guide for how much to eat. Consumers will intend to consume the majority of that portion and have little desire to consume further food (Fig. 1), consistent with a unit bias (Geier et al., 2006). In contrast, we hypothesise that for portions that are perceived as ‘larger than normal’, the intention is likely to be to consume less than the entire portion. For a portion size perceived as ‘smaller than normal’, compensatory eating of additional food may be likely. To test this, participants in each study reported how much of each portion they would intend to consume if it were served to them, which was compared between portion sizes grouped according to their position relative to the norm range. Further, we hypothesise that because portion sizes within the norm range would be treated perceptually similarly by consumers, changes to portion size that occur within the norm range would result in minor changes to intended consumption. In contrast, changes in portion size of the same magnitude that occur across the boundaries of the norm range (e.g., from a ‘normal’ to a ‘smaller than normal’ portion) would result in larger changes to intended consumption.

## 2 Methods

### 2.1 Participants

We calculated that a sample size of 52 would be required to detect small sized effects (*f* = 0.10, correlation between repeated measures = 0.75, non-sphericity correction = 0.75) with 80% power and an alpha level of 0.05 using a 3 (portion size comparison category: across lower norm boundary, within norm range, across upper norm boundary) × 5 (food type) repeated measures ANOVA (G*power, Faul, Erdfelder, Lang, & Buchner, 2007). We decided a priori to recruit 60 participants for Study 1 in order to maintain adequate statistical power in the event of having to exclude participants from analyses due to data loss. We restricted recruitment to adults with a BMI of between 22.5 and 32.5 kg/m^2^, as approximately 70% of adults in England fall within this range (NatCen Social Research, 2016). In addition, recruitment in Study 1 was stratified by gender and two BMI categories (22.5–27.5 kg/m^2^, 27.5–32.5 kg/m^2^, based on self-reported height and weight at recruitment). Participants were recruited from staff and students at the University of Liverpool and from the local community for a study about ‘meal perception’. Individuals with food allergies or intolerances or a history of eating disorders were ineligible, and participants were required to like most everyday foods to be eligible to participate. Participant eligibility was determined using an online screening questionnaire in which participants reported their gender, height, and weight; whether they liked most everyday foods, and whether they had any food allergies, intolerances, or history of eating disorders (yes/no).

For Study 2, a power calculation using G*Power (Faul et al., 2007) indicated that a sample of 34 participants was required to detect the main effect of boundary category (across lower, within, across upper norm range boundaries) on differences in intended consumption observed in Study 1 (η_ρ_^2^ = 0.16, non-sphericity correction = 0.75), with 80% power and alpha set at 0.05. As in Study 1, we over-recruited to allow for data loss. The recruitment strategy and inclusion and exclusion criteria for Study 2 were identical to Study 1, except that the sample was not stratified by gender and BMI category.

No participants from Study 1 were enrolled in Study 2. Both Study 1 and Study 2 received institutional ethical approval (IPHS-1516-LB-243-Generic RETH00095; IPHS-1516-LB-265-Generic RETH000955). All participants provided informed consent and were provided a small monetary reimbursement or course credit for their time.

### 2.2 Materials

#### 2.2.1 Portion size stimuli

Five test foods (porridge, chicken curry with rice, pasta with tomato sauce, chocolate cake with ice-cream, potato crisps) were selected on the basis of a pilot study which indicated that these foods were well-liked and regularly consumed (see online supplemental materials). Portions of porridge, chicken curry, pasta, and crisps varying in 10% size increments from 40% to 300% of the manufacturer’s recommended serving sizes (reference portions, see online supplemental materials for size and product characteristics) were photographed, resulting in a total of 27 images per food. The reference portion of chocolate cake with ice-cream was judged by two authors (AH and ER) to be considerably smaller than what many individuals would judge as ‘normal’. To provide more certainty that the range of portion size stimuli presented to participants would clearly extend beyond what was perceived as ‘normal’, portions of chocolate cake varying in 10% size increments from 40% to 400% of the reference portion were photographed, resulting in a total of 37 images. The pasta, curry, and crisps were presented on standard sized white dinner plates (255 mm diameter), and the porridge and chocolate cake were presented in wide bowls (225 mm diameter, 35 mm depth). All portions were photographed on a white background alongside standard sized cutlery for scale. Photographs were taken using a digital camera positioned at 42⁰ above the horizontal to simulate the average viewpoint from a seated position (following Nelson, Atkinson, & Darbyshire, 1994).

#### 2.2.2 Portion size normality task

In Study 1, participants were presented with a series of images of portion sizes of five different foods, and made a single perceptual judgment of whether they thought each portion was ‘normal’ in size. The ‘norm range’ was identified as the range of portions that were perceived as ‘normal’ by a majority of the sample. As the ‘norm ranges’ in Study 1 were based on the portion sizes perceived as normal by the majority of the sample, it is possible that they did not encompass what was perceived as normal by each individual participant. Therefore in Study 2, participants provided multiple judgments of normality for each portion size which enabled calculation of individual ‘norm ranges’ based on the portion sizes that were judged as being ‘normal’ on the majority of trials by that participant. This approach enabled a conceptual replication of the Study 1’s finding using individually-defined ‘norm ranges’ for a more direct test of the proposed norm range model.

In order to assess portion size normality in each study, participants completed normality judgments for each portion size of each food using a two-alternative forced-choice procedure as in Tovée et al. (2012). The task was programmed using PsychoPy (Peirce, 2007) and presented on a 1280 × 1024 monitor. Each trial consisted of a 250 ms fixation cross, followed by the presentation of a portion size image (1000 × 667 pixels), which remained on screen until the participant made a response. Participants were instructed to categorise the amount of food displayed as either a ‘normal’- or ‘not normal’-sized portion quickly and accurately, by pressing either the left (‘z’), or right (‘m’) key marked on the computer keyboard. The key assigned to each response category was counterbalanced between participants, such that for 50% of participants, the ‘left’ key was used to categorise a portion as ‘normal’ and the ‘right’ key was used to categorise portion as ‘not normal’, and for the other 50% of participants, the key assignment was reversed. During the task, the category labels ‘normal’ and ‘not normal’ were displayed below each portion size image on the side corresponding to the appropriate response key until a response was made. The images were presented in blocks organised by food type (e.g., all images of one food type were viewed consecutively, followed by all images of another food type). The order in which the portion sizes were presented was randomised within each food type block, and the order of presentation of the food type blocks was randomised. Each participant viewed the entire set of portion sizes for all 5 food types once in Study 1, and the entire set of portion sizes for 2 foods 10 times in Study 2. Due to time constraints introduced by increasing the number of normality judgments and the number of computer tasks, participants in Study 2 made judgments of only two foods (pasta, curry). These two foods were chosen as the portion size effect has been most widely studied in main meals (Zlatevska et al., 2014).

#### 2.2.3 Intended consumption task

Intended consumption was assessed in each study using the same methodology as the normality judgment task, except that participants indicated how much of each portion they would plan to eat if it were served to them. Participants indicated their intended consumption for each portion size using the mouse to select a point on a Likert scale positioned below the image. The scale ranged from *1* (*Only a very small part of the portion – it is too big*)*,* to 7 (*The whole portion and a lot more – it is too small*), with a mid-point of 4 (*The whole portion – it is just the right amount*), and remained on screen until a response was made. The intermediate points on the Likert scale were marked with vertical lines but were not labelled. The images were presented in blocks organised by food type (e.g., all images of one food type were viewed consecutively, followed by all images of another food type). The order in which the portion sizes were presented was randomised within each food type block, and the order of presentation of the food type blocks was randomised. Participants completed the normality rating and intended consumption ratings as separate tasks. The randomisation of portion size and food block orders occurred within each task, such that the order was not necessarily the same within each task. Participants provided only one intended consumption judgment for each portion size of each food (5 foods in Study 1, 2 foods in Study 2) in both studies.

#### 2.2.4 Relative size judgment task

In Study 2, we assessed the speed and accuracy of perceptual discrimination between different sized portions using a relative size judgment task. During the task, participants were presented with a series of pairs of portion size images of the same food type, and were asked to judge the relative size of the portions displayed. A set of 25 portion size pairs was generated for each food type, which consisted of each portion size in the stimulus set paired with another that differed by 20% of the reference portion (e.g., 40% vs. 60% portions, 50% vs. 70% portions). The portion size stimuli within each pair therefore differed by the same absolute change in food volume on an interval scale. Each trial presented two images side by side, and participants were asked to indicate which portion size (‘left’ or ‘right’ using the ‘z’ and ‘m’ keys) was largest (smallest). The largest (smallest) portion size image in each pair appeared on each side of the screen with equal frequency. The type of size judgment (‘which is larger’, ‘which is smaller’) was counterbalanced across participants. After participants made a response, the portion size pair remained on screen for 100 ms, before a blank screen with a central fixation cross for 250 ms, followed by the next portion size judgment trial. Each portion size pair was presented 10 times. The order in which the portion sizes were presented was randomised within each food type (pasta, curry), and the order of presentation of the food type blocks was randomised.

### 2.3 Procedures

Participants were instructed not to eat for two hours before attending the session. During the experiment, participants first reported their current level of hunger (7-point Likert scale, anchors: *not at all, extremely*), and how long since they had last eaten. The normality and intended consumption tasks were completed in a counterbalanced order, such that 50% of participants completed the normality task first, and 50% completed the intended consumption task first. Participants then completed a questionnaire assessing demographic characteristics and a standard battery of measures assessing eating habits and preferences (see online supplemental materials). Finally, the researcher measured participants’ height in centimetres using a stadiometer (to 0.1 or 0.5 cm) and weight in kilograms (without shoes and heavy clothing to 0.01 or 0.1 kg) using a digital scale^1^ before debriefing. The procedure for Study 2 was identical to Study 1, except participants completed the relative size judgment task first, followed by the normality and intention rating tasks in a counterbalanced order. The relative size judgment task was completed first to prevent participant responding on the normality task affecting size discrimination. Participants were tested between 9 am and 5:30 pm. Study 1 was conducted between June and August 2016 and Study 2 was conducted between December 2016 and March 2017.

### 2.4 Analysis strategy

#### 2.4.1 Defining the norm range

In Study 1, we used data from the normality judgment task to determine the norm range for each food. For each food the lower boundary of the norm range was identified as the smallest portion size judged as ‘normal’ by a clear majority of participants, and the upper boundary as the largest portion size judged as ‘normal’ by a clear majority of participants. We operationalised a clear majority as being ≥60% of participants (36/60) (see online supplemental materials for justification for this criteria). For the analysis of Study 2, data from the normality task completed in Study 2 was used to determine the norm range for each food for each participant individually. Specifically, for each participant, the proportion of times each portion size was judged as ‘normal’ from the 10 judgments was calculated. The lower boundary of the individual participant norm range for each food was marked by the smallest portion size that was judged as ‘normal’ in ≥60% of trials, and the upper boundary as the largest portion size judged as ‘normal’ in ≥60% of trials. ‘Norm ranges’ based on the portions considered ‘normal’ by a majority of the sample (‘collective norm ranges’) were also calculated for Study 2 in order to directly replicate the findings from Study 1 (see online supplemental materials).

#### 2.4.2 Intended consumption by portion size

In Study 1, we first examined the mean intended consumption of portion sizes falling within, above and below the norm range using a 3 (portion size category: below, within, above norm range) × 5 (food type: cake with ice-cream, crisps, curry with rice, pasta, porridge) repeated measures ANOVA. We conducted the same analysis in Study 2 using individually-defined norm ranges across the 2 foods (pasta, curry). As intended consumption was expressed as an amount relative to the whole portion, we expected lower intended consumption from portions above the norm range (i.e., intention to eat only a small amount of the portion) than from portions within the norm range (i.e., intention to consume the entire portion but no more), and the highest intended consumption from portions that were below the norm range (i.e., intention to consume the entire portion plus more, or ‘intended compensation’). In addition, we conducted a series of one-sample *t*-tests to compare the mean intended consumption within each category (below, within, above norm) with the midpoint of the scale (4).

#### 2.4.3 Sensitivity of intended consumption to category boundaries

Next, we tested the hypothesis that intended consumption would be more sensitive to changes in portion size that occur across the boundaries of the norm range relative to within the norm range. First, the size of the difference in intended consumption between pairs of portions that differed by size increments equal to 20% of the reference portion were calculated for each food type (e.g., 50% versus 70%, 60% versus 80% portions, representing the same absolute change in food volume on an interval scale; see online supplemental materials for further information on this criterion). Then, the mean of these size difference scores were calculated for portion size pairs that were positioned (a) across the lower norm range boundary, (b) within the norm range, and (c) across the upper norm range boundary. Larger differences reflected greater sensitivity of intended consumption to changes in portion size at the respective position in the norm range. Differences in intended consumption were compared in a 3 (comparison: across lower boundary, within norm range, across upper boundary) × 5 (food type) repeated measures ANOVA (×2 food types in Study 2).

#### 2.4.4 Categorical perception of portion size normality

To assess evidence for the categorical perception of portion size, we tested the hypothesis that participants would be better able to discriminate between pairs of portion sizes crossing the boundaries of the norm range than portion sizes that were contained within the norm range (Study 2). Discrimination performance was operationalised as relative size judgment reaction time (RT) and accuracy. Relative size judgment trials with a RT <350 ms and >3SD from the sample mean (4097 ms) were first discarded to eliminate anticipatory and outlying responses (Tovée et al., 2012, van Selst and Jolicoeur, 1994). This trimming procedure resulted in a data loss of 2.12%, and the pattern of results remained the same when untrimmed RTs were analysed. We also conducted a sensitivity analysis using natural log-transformed values to correct skewness in the RT data. The pattern of results remained the same despite transformation. Results from analysis of untransformed data are reported for interpretability. The mean RT of correct judgments, and accuracy (proportion correct) of relative size judgments in response to each relative size judgment trial were calculated, with lower RT and higher accuracy indicating superior discrimination performance. The mean RT and accuracy were then calculated for relative size judgment trials that were positioned (a) across the lower norm range boundary, (b) within the norm range, and (c) across the upper norm range boundary. Relative size judgment RT and accuracy were compared using separate 3 (comparison position: across lower boundary, within norm range, across upper boundary) × 2 (food type) repeated measures ANOVAs.

Weber’s law holds that as the size of a physical stimulus increases or decreases, discrimination performance diminishes or improves (Kingdom & Prins, 2016). Therefore, differences between smaller portion sizes should be easier to discriminate than differences between larger portion sizes. Because comparison position in the relative size judgment task (across lower boundary, within norm range, across upper boundary) is confounded with portion size, effects on discrimination performance could also be attributable to Weber’s law and not only categorical perception. We therefore conducted further post-hoc analyses to consider this possibility (described in full in the online supplemental materials).

#### 2.4.5 Adjustment for multiple comparisons and effect sizes

One-sample *t*-tests were considered significant at *p* < .003 for Study 1 (α = 0.05/κ, κ = 15 comparisons), and *p* < .008 for Study 2 (α = 0.05/κ, κ = 6 comparisons). Significant main effects of food type in each two-way ANOVA were followed up with pairwise comparisons which were considered significant at *p* < .0036 for Study 1 (κ = 14) and *p* < .05 for Study 2 (κ = 1). Significant interactions in each analysis were followed up with subsequent one-way repeated-measures ANOVAs analysing the effect of portion size (or norm range boundary category) on the dependent variable (intended consumption, mean difference intended consumption, relative size judgment RT/accuracy) within each food type. Significant main effects of portion size or boundary category were followed up with pairwise comparisons which were considered significant at *p* < .025 for Study 2 (κ = 2). Effect sizes are reported as partial eta squared (η_ρ_^2^) (small = 0.01, medium = 0.06, large = 0.14, Cohen, 1988).

## 3 Results

The recruited sample in Study 1 consisted of 60 participants (50% women) aged between 21 and 73 years (*M* = 40.33, *SD* = 14.90) with a measured BMI range of 20.27 to 36.15 kg/m^2^ (*M* = 27.35*, SD* = 3.43). All participants were included in analyses. In Study 2, the recruited sample consisted of 46 participants (74% female) aged between 18 and 48 years (*M* = 21.00, *SD =* 4.65), with measured BMI ranging 18.87–33.99 kg/m^2^ (*M* = 23.80, *SD* = 3.18). The analytic sample size for the individual norm range analyses in Study 2 ranged from 29 to 37 (see online supplemental materials for details on missing data).

### 3.1 Portion size ‘norm ranges’

In Study 1, for all 5 foods we found that there were a wide range of portion sizes that a clear majority of participants categorised as being ‘normal’. As a percentage of the reference portion, the norm range for porridge was 100–150%, for pasta was 70–120%, for curry was 80–160%, for crisps was 130–190%, and for chocolate cake and ice-cream was 90–170%. Results for the collective norm ranges in Study 2 are discussed in the online supplemental materials.

### 3.2 Effect of portion size on intended consumption

As predicted, there was a main effect of portion size (below, within, above norm range) on intended consumption, Study 1: *p* < .001, η_ρ_^2^ = 0.95, Study 2: *p* < .001, η_ρ_^2^ = 0.95 (see Table S2 for complete ANOVA results). Participants reported greater intended consumption for portion sizes below the norm range (where they intended to eat the entire portion plus more) than within the norm range (where they intended to eat just the displayed portion), Study 1: mean difference (*MD*) intended consumption = 1.63, SE = 0.06, *p* < .001, Study 2: *MD* = 1.81, SE = 0.10, *p* < .001. Participants also reported greater intended consumption from portions from within the norm range (where they intended to eat just the displayed portion) than above the norm range (where they intended to eat only a part of the portion), Study 1: *MD* = 1.68, SE = 0.05, *p* < .001, Study 2: *MD* = 1.78, SE = 0.09, *p* < .001. There was no significant main effect of food type, but a significant interaction between portion size and food type in both Study 1: <0.001, η_ρ_^2^ = 0.33, and Study 2: *p* < .05, η_ρ_^2^ = 0.10. Follow-up one-way repeated measures ANOVAs confirmed that portion size predicted intended consumption for each food type in both studies (Table 1).

**Table 1:** Intended consumption between portion size categories.

Food	Below norm	Within norm	Above norm	η_ρ_^2^(*n*)^a^
Study 1				
Chocolate cake	5.75 (0.87)*	4.08 (0.78)	2.07 (0.60)*	0.93 (60)
Curry	5.73 (0.75)*	3.78 (0.71)	2.04 (0.85)*	0.93 (60)
Crisps	5.29 (0.92)*	3.58 (1.12)	2.56 (1.11)*	0.91 (60)
Pasta	5.49 (1.03)*	4.17 (0.97)	2.12 (0.86)*	0.91 (60)
Porridge	5.47 (0.97)*	3.99 (0.82)	2.42 (0.72)*	0.89 (60)

Study 2				
Curry	5.90 (0.68)*	3.98 (0.53)	2.20 (0.64)*	0.94 (41)
Pasta	5.68 (0.75)*	3.96 (0.68)	2.23 (0.70)*	0.94 (31)

*Note.* Values are mean (standard deviations in parentheses) intended consumption ranging from *1* (*Only a very small part of the portion – it is too big*) to 7 (*The whole portion and a lot more – it is too small*), with a midpoint of 4 (*The whole portion – it is just the right amount*).

* Statistically significant one-sample *t*-test comparing mean intended consumption with test value of 4.

a From repeated-measures ANOVAs comparing portion size categories. All ANOVAs and pairwise comparisons within food types significant at *p* < .001.

For all foods across both studies, one-sample *t*-tests indicated that the mean intended consumption was significantly below the midpoint of the scale for portions above the norm range (indicating that participants intended to consume only a part of the portion), and significantly above the midpoint of the scale for portions below the norm range (indicating that participants intended to compensate by eating more than the displayed portion). Mean intended consumption did not significantly differ from the midpoint of the scale for portions within the norm range (indicating that participants intended to consume the entire portion and no more, Table 1).

The same pattern of results in Study 2 was observed using collective ‘norm ranges’, directly replicating the findings from Study 1. Intended consumption of both foods was significantly higher for portions below the norm range than within the norm range, and for portions within the norm range than for above the norm range (see online supplemental materials).

### 3.3 Sensitivity of intended consumption to portion size norm range boundaries

**Fig. 2:**
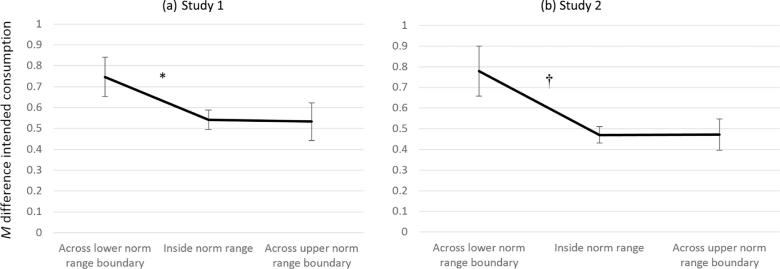
a and b. Mean difference in intended consumption between pairs of portion sizes grouped by norm boundary position for Study 1 (a) and Study 2 (b). Larger mean differences indicate greater sensitivity of intended consumption to changes in portion size at the respective position in the norm range. * indicates a significant difference (*p* < .025) between adjacent norm boundary categories. † indicates *p* = .025. Error bars represent standard error of the mean.

In both studies, boundary category (across lower, within, across upper norm range boundaries) predicted differences in intended consumption between pairs of portions differing by 20% size increments, Study 1: *p* < .001, η_ρ_^2^ = 0.16 (Fig. 2a), Study 2: *p* = .03, η_ρ_^2^ = 0.11 (Fig. 2b). As predicted, pairwise comparisons revealed the mean difference in intended consumption was significantly larger between portion sizes in the pair that crossed the lower norm boundary than between portion size pairs that fell inside of the norm range, Study 1: *MD* difference in intended consumption between paired portion sizes = 0.20, SE = 0.05, *p* < .001, but this comparison was only borderline significant (against Bonferroni-adjusted α = 0.025) in Study 2, *MD* = 0.31, SE = 0.13, *p* = .025. Contrary to predictions, mean differences in intended consumption did not differ between portion sizes that fell within the norm boundary and those in the pair that crossed the upper norm boundary in either Study 1, *MD* = 0.01, SE = 0.05, *p* = .99, or Study 2, *MD* = −0.001, SE = 0.08, *p* = .99. Across both studies, type of food predicted differences in intended consumption between portion size pairs, Study 1: *p* = <0.001, η_ρ_^2^ = 0.08, Study 2: *p* = .033, η_ρ_^2^ = 0.13, but there was no significant interaction between food and boundary category.

Using ‘collective norm ranges’ in Study 2, the mean difference in intended consumption tended to be larger between portion sizes that crossed the lower norm boundary than between portion sizes that fell inside the norm range, although this was not statistically significant (*p* = .047, against Bonferroni-adjusted α = 0.025). In addition, the mean difference in intended consumption was significantly larger between portion sizes that crossed the upper norm range boundary than within the norm range boundary (see online supplemental materials).

### 3.4 Effect of norm range on discrimination performance

**Fig. 3:**
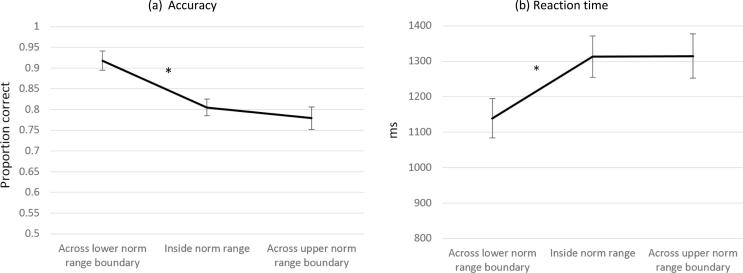
a and b. Relative size judgment performance (accuracy [a], reaction time [b]) by norm boundary position. Greater accuracy and lower (faster) reaction time indicate better discriminability of relative portion sizes at the respective position in the norm range. * indicates a significant difference (*p* < .025) between adjacent norm range boundary categories. Error bars represent standard error of the mean.

In Study 2, the position of portion size pairs relative to the norm range boundary category (across lower, within, across upper norm range boundaries) predicted discrimination performance (relative size judgment accuracy (Fig. 3a), *p* < .001, η_ρ_^2^ = 0.43, and RT, (Fig. 3b), *p* < .001, η_ρ_^2^ = 0.34). Neither food type nor the interaction between food type and boundary category predicted accuracy. In line with predictions, pairwise comparisons revealed that relative size judgments were more accurate, *MD* proportion correct = 0.11, SE = 0.01, *p* < .001, and faster, *MD* ms = −174.21, SE = 26.71, *p* < .001, for the portion pairs that crossed the lower norm boundary than between portion size pairs that fell inside the norm range. However, neither accuracy, *MD* = 0.03, SE = 0.02, *p* = .20, nor RT, *MD* = −1.38, SE = 31.51, *p* = .97, significantly differed between portion size pairs that fell within the norm range boundary and the pairs that crossed the upper norm boundary. There was a significant interaction between food type and boundary category in predicting relative size judgment reaction time, *p* = .003, η_ρ_^2^ = 0.17. Consistent with predictions, pairwise comparisons revealed that reaction times were faster for the portion sizes pair that crossed the lower norm boundary than between portion size pairs that fell within the norm range for both foods, curry: *MD* = −212.30, SE = 34.20, *p* < .001, pasta: *MD* = −105.03, SE = 30.88, *p* = .002 (against Bonferroni-adjusted α = 0.025). However, mean RT did not significantly differ between portion size comparisons that fell within the norm range boundary and the pair that crossed the upper norm boundary, curry: *MD* = −12.59, SE = 31.52, *p* = .69, pasta: *MD* = −43.02, SE = 45.92, *p* = .36. As the pattern of results is consistent across foods, mean reaction times between boundary categories for curry and pasta combined are plotted in Fig. 3b.

We conducted post-hoc analyses to explore the relative roles that categorical perception and Weber’s law have in explaining why relative size of portion sizes that crossed the lower norm boundary were judged more quickly and accurately than portions within the norm range (see online supplemental materials). The results were suggestive that although categorical perception was in part responsible for this tendency (consistent evidence for one of the foods, mixed evidence for the other), the pattern of results appeared to be largely attritubable to Webers’ law.

## 4 Discussion

Across two studies, we provide evidence that perceptions of portion size normality predict intended food consumption. In line with our predictions, both studies demonstrated that for most foods, portions within the range considered ‘normal’ in size were intended to be consumed in their entirety without compensation. In contrast, participants indicated that they would intend to ‘compensate’ for portions that were considered ‘smaller than normal’, and that they they would consume only part of the entire portion of food when that portion was considered ‘larger than normal’. Importantly, in both studies, we found that intended consumption was more sensitive to changes in portion size that occurred across the lower boundary of the norm range than changes in portion size of the same magnitude that occurred within the norm range. This suggests that altering portion size does not influence intended consumption in a purely linear manner, but rather that intended consumption is sensitive to the difference between perceptual categories of ‘normal’ versus ‘not normal’ portion sizes. A speculative explanation is that this could be attributable to the notion that humans have evolved to possess physiological and psychological mechanisms that protect against low adiposity (Berthoud, 2004, Blundell and Gillett, 2001, Zheng and Berthoud, 2008). Consuming an amount of food that exceeds immediate physiological needs would have been adaptive to buffer against periods of food scarcity that were common until very recently in our evolutionary history (Berthoud, 2004, Blundell and Gillett, 2001, Pinel et al., 2000, Zheng and Berthoud, 2008). Therefore, consumers may be sensitive to detecting an insufficiency of food (e.g., a ‘smaller than normal’ portion size).

The finding that differences in intended consumption were no more sensitive to changes occurring across the upper boundary of the norm range relative to within the norm range could explain why energy intake continues to increase in response to portion sizes that are already very large (e.g., Kral et al., 2004, Rolls et al., 2006a). Although the portion size effect is curvilinear and begins to plateau at portion sizes of large magnitude, this trend is gradual, and significant increases in intake are still observed with increases to very large portions (Zlatevska et al., 2014). We speculate that there will be a point at which increasing portion size no longer results in an increase in energy intake, but contrary to our initial predictions this limit may be set primarily by physiological constraints (i.e., by how much can comfortably be ingested) rather than by perceptions of portion size normality. Testing the point at which an increase in portion size no longer results in an increase in energy intake in an experiment with actual food consumption would be informative.

In primary analyses of the relative size judgment task, we only found evidence of better discrimination between ‘normal’ and ‘smaller than normal’ portion sizes relative to portions within the norm range, and not between ‘normal’ and ‘larger than normal’ portion sizes. We believe these results are primarily explained by a basic perceptual bias, Weber’s law (Kingdom & Prins, 2016). According to Weber’s law, the same absolute differences in portion size (i.e., a change that is equal in terms of food volume) should become progressively more difficult to discriminate as portion size increases (e.g. moving from smaller than normal to normal portion size judgements). This is consistent with previous research showing that estimation of product sizes becomes more inaccurate as size increases (Chandon and Ordabayeva, 2009, Ordabayeva and Chandon, 2016). Results of our additional analyses were illustrative of Weber’s law: discrimination performance on the relative size judgement task became progressively worse with increasing portion size. However, this was not the only pattern describing discrimination performance. We also observed some change in discrimination performance at the categorical boundaries of the ‘norm range’, which is suggestive of categorical perception. Taken together, the present findings suggest that although there are a wide range of portion sizes perceived as being ‘normal’ in size by consumers and perceived normality of portion size is predictive of intended consumption, it is less clear whether this specific range of portion sizes is treated by the visual system as a separate perceptual category. Further work designed to specifically address this will be required. Specifically, future research could present portion stimuli that vary by the same relative amount at each increment (e.g., reduction of 10% of each portion size) rather than the same absolute amount (e.g., a reduction by the same volume of food). This would provide an alternative approach to testing whether portion size normality is categorically perceived that is not reliant on the stimulus arrangement used in the present studies.

Reducing the portion size of commercially available foods has been suggested as a potential public health strategy to reduce overconsumption and tackle obesity (Livingstone and Pourshahidi, 2014, Marteau et al., 2015). Our findings using intended food consumption suggest that portion size reductions may be most effective at reducing overall energy intake when they are gradual, and more specifically, when the resultant portion sizes are still considered to be ‘normal’ in size in order to minimise compensatory eating. It is possible that when a portion is reduced to the point where it is perceived as being ‘smaller than normal’, overall intake may equal or even exceed what would have been consumed from a larger portion because of the compensatory eating that could occur. Therefore, rather than a portion size reduction of this kind being ineffective at reducing intake, it may even backfire and increase intake. However, a series of studies have now shown that participants who were visually exposed to smaller portions of food perceived smaller portions of food as more appropriate or ‘normal’ (Robinson et al., 2016). It follows then, that reductions to portion size of foods may feed back into perceived norms by shifting the range of portion sizes perceived as ‘normal’ downwards. This in turn may enable another gradual reduction within this new ‘norm range’. Investigating how long it takes for portion size norms to adjust in response to environmental changes would be valuable in planning a gradual and stepwise reduction to commercially available portion sizes to reduce overeating. In addition, only a small number of studies have examined the effect of reducing portion sizes on energy intake (French et al., 2014, Lewis et al., 2015, Rolls et al., 2006b), and it is unclear how the portion sizes in these studies were perceived by participants. It would now be informative to examine whether the results we observed here for intended consumption translate to actual compensation and more specifically, whether perceptions of portion size normality determine when compensatory eating occurs in response to reductions in portion size.

Previous research has shown that exposure to smaller (versus larger) portion sizes results in smaller portion sizes being perceived as normal, suggesting that perceived normality is driven by one’s ‘visual diet’ (Robinson et al., 2016). A second (and related) potential driver of perceived portion size normality may be one’s prior eating experiences. The central premise of the norm range model is that perceived normality drives how much of a given portion will be eaten, but the relationship between perceived normality of a given portion size and consumption may be bidirectional. That is, the portion sizes that are perceived as ‘normal’ are also likely to be determined in part by how much an individual usually consumes of that food in a single eating occasion. By extension, perceived portion size normality may therefore be in part influenced by other factors associated with energy intake and portion size selection such as early childhood experiences, gender, weight status, and motivational factors such as dietary restraint (Almiron-Roig et al., 2018, Brunstrom et al., 2008, Burger et al., 2007, Lewis et al., 2015, McCrickerd and Forde, 2016, Rolls et al., 1991, Spence et al., 2016). We found evidence in both studies that a relatively wide range of portion sizes of all foods were perceived as being ‘normal’ in size by the majority of participants and despite subtle differences in the exact location of the norm range based on majority responses in Studies 1 and 2, there was considerable overlap between the norm ranges in both studies. It was not our aim in the present work to examine whether participant characteristics are associated with the range of portion sizes perceived as being ‘normal’ in size (see Lewis et al., 2015, Spence et al., 2016, Zlatevska and Spence, 2016) and further research specifically designed to address this question would be of value.

The present research has several strengths and limitations. A strength of the research was that consistent findings emerged across two independent samples and five food types. However, the foods tested were mostly amorphous, and investigation of whether the predictions of the norm range model are supported in foods with a small number of discrete units (e.g., pizza slices, biscuits) is needed. It has been suggested that personal portion size norms and estimation of portion size may be influenced by food-related factors such as healthiness, energy density, unit size, and manner of presentation, including plate size (Almiron-Roig et al., 2013, Penaforte et al., 2014, Zlatevska and Spence, 2016). It may now be of interest to investigate whether the predictions of the norm range model are supported when food stimuli are systematically varied along these dimensions. It is important to note that the approach adopted in the present studies does not allow us to make causal inferences about the influence that portion size normality has on intended consumption, although previous work has shown that manipulating the perceived normality of a portion size affects how much of that food a consumer would intend to eat in future (Robinson et al. 2016). A limitation of Study 1 is that the maximum portion size of chocolate cake and ice cream was larger than for other foods. As visual exposure to larger or smaller portion sizes can bias what is perceived as normal (Robinson et al., 2016), it is possible that the inclusion of a larger range of portion sizes of chocolate cake resulted in an upwards shift of the norm range for this food. However we note that the pattern of results for each analysis in Study 1 was consistent across food types.

The norm range model of the portion size effect was supported across two studies examining intended consumption of different portion sizes. Results suggest that reductions to portion size where the resultant portion size is considered ‘smaller than normal’ may not significantly reduce energy intake because individuals may intend to engage in compensatory eating. However, reductions which result in the reduced portion size being considered ‘normal’ are likely to reduce energy intake without inviting intended compensatory eating. These conclusions are based on self-reported intended food consumption and therefore, further research examining whether the influence that portion size has on actual food consumption can be explained through a norm range model is required.

## Footnotes

1 In Study 1 and the first 14 participants in Study 2, participant height was recorded to the nearest 0.1 cm. For the remainder of participants in Study 2, height was recorded either to the nearest 0.1 or 0.5 cm. Inconsistencies in height recording are due to researcher error. Weight was recorded to the nearest 0.01 kg for the first 24 participants in Study 1 and to the nearest 0.1 kg for the remainder of participants in Study 1 and 2, due to a change in equipment.

## References

[b0005] Almiron-Roig E., Navas-Carretero S., Emery P., Martínez J.A. (2018). Research into food portion size: Methodological aspects and applications. Food & Function.

[b0010] Almiron-Roig E., Solis-Trapala I., Dodd J., Jebb S.A. (2013). Estimating food portions. Influence of unit number, meal type and energy density. Appetite.

[b0015] Berthoud H.-R. (2004). Mind versus metabolism in the control of food intake and energy balance. Physiology & Behavior.

[b0020] Blundell J.E., Gillett A. (2001). Control of food intake in the obese. Obesity Research.

[b0025] Brunstrom J.M., Rogers P.J., Pothos E.M., Calitri R., Tapper K. (2008). Estimating everyday portion size using a ‘method of constant stimuli’: In a student sample, portion size is predicted by gender, dietary behaviour, and hunger, but not BMI. Appetite.

[b0030] Bucher T., Rollo M.E., Smith S.P., Dean M., Brown H., Sun M., Collins C. (2017). Position paper on the need for portion-size education and a standardised unit of measurement. Health Promotion Journal of Australia.

[b0035] Burger K.S., Kern M., Coleman K.J. (2007). Characteristics of self-selected portion size in young adults. Journal of the American Dietetic Association.

[b0040] Calder A.J., Young A.W., Perrett D.I., Etcoff N.L., Rowland D. (1996). Categorical perception of morphed facial expresions. Visual Cognition.

[b0045] Chandon P., Ordabayeva N. (2009). Supersize in one dimension, downsize in three dimensions: Effects of spatial dimensionality on size perceptions and preferences. Journal of Marketing Research.

[b0050] Cohen J. (1988). Statistical power analysis for the behavioral sciences.

[b0055] Diliberti N., Bordi P.L., Conklin M.T., Roe L.S., Rolls B.J. (2004). Increased portion size leads to increased energy intake in a restaurant meal. Obesity Research.

[b0060] Faul F., Erdfelder E., Lang A.-G., Buchner A. (2007). G*Power 3: A flexible statistical power analysis program for the social, behavioral, and biomedical sciences. Behavior Research Methods.

[b0065] French S.A., Mitchell N.R., Wolfson J., Harnack L.J., Jeffery R.W., Gerlach A.F., Pentel P.R. (2014). Portion size effects on weight gain in a free living setting. Obesity.

[b0070] Geier A.B., Rozin P., Doros G. (2006). Unit bias: A new heuristic that helps explain the effect of portion size on food intake. Psychological Science.

[b0075] Harnad S., Harnad S. (1987). Psychophysical and cognitive aspects of categorical perception: A critical overview. Categorical perception: The groundwork of cognition.

[b0080] Herman C.P., Polivy J. (1983). A boundary model for the regulation of eating. Psychiatric Annals.

[b0085] Herman C.P., Polivy J. (2005). Normative influences on food intake. Physiology & Behavior.

[b0090] Hollands G.J., Shemilt I., Marteau T.M., Jebb S.A., Lewis H.B., Wei Y., Ogilvie D. (2015). Portion, package or tableware size for changing selection and consumption of food, alcohol and tobacco. Cochrane Database of Systematic Reviews.

[b0095] Kelly M.T., Wallace J.M., Robson P.J., Rennie K.L., Welch R.W., Hannon-Fletcher M.P., Livingstone M.B. (2009). Increased portion size leads to a sustained increase in energy intake over 4 d in normal-weight and overweight men and women. British Journal of Nutrition.

[b0100] Kerameas K., Vartanian L.R., Herman C.P., Polivy J. (2015). The effect of portion size and unit size on food intake: Unit bias or segmentation effect?. Health Psychology.

[b0105] Kingdom F.A.A., Prins N. (2016). Psychophysics: A practical introduction.

[b0110] Kral T.V., Roe L.S., Rolls B.J. (2004). Combined effects of energy density and portion size on energy intake in women. The American Journal of Clinical Nutrition.

[b0115] Lapid E., Ulrich R., Rammsayer T. (2008). On estimating the difference limen in duration discrimination tasks: A comparison of the 2AFC and the reminder task. Perception & Psychophysics.

[b0120] Lapid E., Ulrich R., Rammsayer T. (2009). Comparisons of two variants of the method of constant stimuli for estimating difference thresholds. Swiss Journal of Psychology.

[b0125] Lawrence D.H. (1950). Acquired distinctiveness of cues: II. Selective association in a constant stimulus situation. Journal of Experimental Psychology.

[b0130] Lewis H.B., Ahern A.L., Solis-Trapala I., Walker C.G., Reimann F., Gribble F.M., Jebb S.A. (2015). Effect of reducing portion size at a compulsory meal on later energy intake, gut hormones, and appetite in overweight adults. Obesity.

[b0135] Lewis H.B., Forwood S.E., Ahern A.L., Verlaers K., Robinson E., Higgs S., Jebb S.A. (2015). Personal and social norms for food portion sizes in lean and obese adults. International Journal of Obesity.

[b0140] Livingstone M.B.E., Pourshahidi L.K. (2014). Portion size and obesity. Advances in Nutrition: An International Review Journal.

[b0145] Logan J.S., Lively S.E., Pisoni D.B. (1991). Training Japanese listeners to identify English/r/and/l: A first report. The Journal of the Acoustical Society of America.

[b0150] Marteau T.M., Hollands G.J., Shemilt I., Jebb S.A. (2015). Downsizing: Policy options to reduce portion sizes to help tackle obesity. BMJ.

[b0155] McCrickerd K., Forde C.G. (2016). Parents, portions and potential distortions: Unpicking children’s meal size. Nutrition Bulletin.

[b0160] NatCen Social Research. (2016). Health Survey for England, 2014 [data collection].

[b0165] Nelson M., Atkinson M., Darbyshire S. (1994). Food photography I: The perception of food portion size from photographs. British Journal of Nutrition.

[b0170] Ng M., Fleming T., Robinson M., Thomson B., Graetz N., Margono C., Abera S.F. (2014). Global, regional, and national prevalence of overweight and obesity in children and adults during 1980–2013: A systematic analysis for the Global Burden of Disease Study 2013. The Lancet.

[b0175] Nielsen S.J., Popkin B.M. (2003). Patterns and trends in food portion sizes, 1977–1998. Journal of the American Medical Association.

[b0180] Nørnberg T.R., Houlby L., Jørgensen L.N., He C., Pérez-Cueto F. (2014). Do we know how much we put on the plate? Assessment of the accuracy of self-estimated versus weighed vegetables and whole grain portions using an Intelligent Buffet at the FoodScape Lab. Appetite.

[b0185] Ordabayeva N., Chandon P. (2013). Predicting and managing consumers’ package size impressions. Journal of Marketing.

[b0190] Ordabayeva N., Chandon P. (2016). In the eye of the beholder: Visual biases in package and portion size perceptions. Appetite.

[b0195] Peirce J.W. (2007). Psychophysics software in Python. Journal of Neuroscience Methods.

[b0200] Penaforte F., Japur C., Diez-Garcia R., Hernandez J., Palmma-Linares I., Chiarello P. (2014). Plate size does not affect perception of food portion size. Journal of Human Nutrition and Dietetics.

[b0205] Pinel J.P.J., Assanand S., Lehman D.R. (2000). Hunger, eating, and ill health. American Psychologist.

[b0210] Robinson E., Oldham M., Cuckson I., Brunstrom J.M., Rogers P.J., Hardman C.A. (2016). Visual exposure to large and small portion sizes and perceptions of portion size normality: Three experimental studies. Appetite.

[b0215] Robinson E., te Raa W., Hardman C.A. (2015). Portion size and intended consumption. Evidence for a pre-consumption portion size effect in males?. Appetite.

[b0220] Rolls B.J., Fedoroff I.C., Guthrie J.F. (1991). Gender differences in eating behavior and body weight regulation. Health Psychology.

[b0225] Rolls B.J., Roe L.S., Meengs J.S. (2006). Larger portion sizes lead to a sustained increase in energy intake over 2 days. Journal of the American Dietetic Association.

[b0230] Rolls B.J., Roe L.S., Meengs J.S. (2006). Reductions in portion size and energy density of foods are additive and lead to sustained decreases in energy intake. American Journal of Clinical Nutrition.

[b0235] Rozin P., Ashmore M., Markwith M. (1996). Lay American conceptions of nutrition: Dose insensitivity, categorical thinking, contagion, and the monotonic mind. Health Psychology.

[b0240] Smiciklas-Wright H., Mitchell D.C., Mickle S.J., Goldman J.D., Cook A. (2003). Foods commonly eaten in the United States, 1989–1991 and 1994–1996: Are portion sizes changing?. Journal of the American Dietetic Association.

[b0245] Spence M., Livingstone M.B.E., Hollywood L.E., Gibney E.R., O’Brien S.A., Pourshahidi L.K., Dean M. (2013). A qualitative study of psychological, social and behavioral barriers to appropriate food portion size control. International Journal of Behavioral Nutrition and Physical Activity.

[b0250] Spence M., Stancu V., Dean M., Livingstone M.B.E., Gibney E.R., Lähteenmäki L. (2016). Are food-related perceptions associated with meal portion size decisions? A cross-sectional study. Appetite.

[b0255] Tovée M.J., Edmonds L., Vuong Q.C. (2012). Categorical perception of human female physical attractiveness and health. Evolution and Human Behavior.

[b0260] van Selst M., Jolicoeur P. (1994). A solution to the effect of sample size on outlier elimination. The Quarterly Journal of Experimental Psychology.

[b0265] Wrieden W., Gregor A., Barton K. (2008). Have food portion sizes increased in the UK over the last 20 years?. The Proceedings of the Nutrition Society.

[b0270] Zheng H., Berthoud H.-R. (2008). Neural systems controlling the drive to eat: Mind versus metabolism. Physiology.

[b0275] Zlatevska N., Dubelaar C., Holden S.S. (2014). Sizing up the effect of portion size on consumption: A meta-analytic review. Journal of Marketing.

[b0280] Zlatevska N., Spence M.T. (2016). Parsing out the effects of personal consumption norms and industry influences on food consumption volume. European Journal of Marketing.

