# Supplementary material for: Visual perceptions of portion size normality and intended food consumption: A norm range model
Source: Food Qual Prefer. 2019 Mar;72:77–85. doi: 10.1016/j.foodqual.2018.10.003 (PMC6333281; doi:10.1016/j.foodqual.2018.10.003)
Supplement: Supplementary data 1 [file mmc1.pdf]

Online Supplemental Materials

Methods

Portion size stimuli

To select the five foods used in Study 1, participants completed an online study in which they answered questions about commonly eaten and commercially available food types. Foods containing a small number of discrete units per portion (e.g., biscuits, chocolate bars) were not included in order to prevent unit size or number from influencing normality judgments. A pilot sample of 22 participants (11 males, 11 females) aged between 20 and 60 years (*M* = 30.33, *SD* = 10.95) rated on 7-point Likert scales how regularly they consumed each of the candidate foods (from 1 = daily, to 7 = yearly or less frequently), their liking of the food, familiarity, and appropriateness of consumption for breakfast, lunch, dinner, dessert, and a snack. We selected foods which were generally well liked and regularly consumed by participants in the pilot study (Table S1).

Table S1.

Portion size stimuli: pilot ratings and product characteristics

Food	Pilot ratings *m (sd)*		Product	Reference portion (manufacturer's recommended serving)	
	Liking	Consumption frequency		g	kcal
Chocolate cake and ice-cream	5.73 (1.45)	5.23 (1.02)	Tesco chocolate fudge cake	75	270
	5.91 (1.12)	5.14 (1.13)	Tesco soft scoop vanilla ice-cream	45	79
			Total	120	349
Curry with rice	5.86 (1.17)	4.23 (1.15)	Tesco chicken curry	200	179
			Tesco microwave long grain rice	125	209
			Total	325	388
Crisps	5.09 (1.34)	4.27 (1.75)	Tesco ready salted crisps	25	136
Pasta with tomato sauce	5.09 (1.48)	4.68 (1.36)	Tesco quick cook penne [a]	170	263
			Tesco everyday value pasta sauce	110	36
			Total	280	299
Porridge	4.77 (1.51)	4.59 (2.20)	Quaker Golden Syrup Porridge [a]	216	215

[a] Prepared according to manufacturers' instructions.

[b] Prepared with 180mL semi skimmed milk.

Criteria for identifying the 'norm range'

For each food, the lower boundary of the norm range was identified as the smallest portion size judged as 'normal' by a clear majority of participants, and the upper boundary as the largest portion size judged as 'normal' by a clear majority of participants. We operationalised a clear majority as being ≥60% of participants. We initially planned to use a criteria of 50% of participants judging a portion size as 'normal' to identify the norm range in Study 1 (based on Tovée, Edmonds, & Vuong, 2012). However, for a number of foods there were portion sizes which clustered around 50%, suggesting a lack of consensus. We therefore increased the norm range criteria to 60% of participants judging a portion size as 'normal', in order to identify a range of portion sizes that was clearly considered normal by a majority of participants, and to differentiate from chance expectation (50%). This criteria was determined before hypothesis testing in Study 1 and was then used in the a priori selection of norm ranges in Study 2.

Calculation of differences in intended consumption

We calculated differences in intended consumption between pairs of portions that differed in size by 20% of the reference portion to allow a 'gap' between paired portion sizes. As participants reported intended consumption of portions sizes differing by 10% size increments, the 20% difference scores meant that each cross-boundary comparison would be expected to more reliably encompass a portion that was perceived by most participants as 'normal' and one that was 'not normal'. For example, if the 80% portion is considered the start of the norm range, a 20% difference score of 60% - 80% provides more certainty that the comparison represents a cross-boundary comparison for the majority of participants (i.e., includes a 'smaller than normal' versus 'normal' comparison) than does a 10% difference score of 70 – 80%.

Individual differences measures

Participants completed a standard battery of questionnaires that we routinely collect in laboratory studies to provide data for future exploratory analyses. See below. We did not intend to use these measures for analytical purposes in the planned analyses for Studies 1 and 2, and do not discuss these variables further in the present work.

Income and education

Participants were asked to indicate their highest level of education *(did not complete high school, high school, some university, Bachelor's degree, Master's degree, Doctoral or professional degree*), and to enter their total annual household income (free text entry).

Pilot check questions

Participants were asked to report how regularly they consume (Likert scale ranging from 1 [*on a daily basis*], to 7 [*yearly or less frequently*], reverse coded), how familiar they are with (from 1 [*not at all familiar*], to 7 [*extremely familiar*]), and how much they like (from 1 [*not at all*], to 7 [*like it a lot*]), each of the test foods.

Restrained, external, and emotional eating

Participants completed the commonly used Dutch Eating Behaviour Questionnaire (DEBQ; Van Strien et al., 1986), which consists of 33 items in three subscales measuring restrained, external, and emotional eating tendencies. The scales have been demonstrated to have good internal reliability (Van Strien et al., 1986).

Dieting status

A single item was administered to assess whether participants were currently dieting (yes/no).

Plate clearing tendency

A 5-item measure with a 5-point Likert response format (1 [*strongly disagree*], to 5 [*strongly agree*]) was used to assess participants' tendencies to clear their plate when eating (e.g., " I always clear my plate when eating"; Robinson et al., 2015). The scale has been demonstrated to have good internal reliability (Robinson et al., 2015).

Self-control

The brief self-control scale was used to assess trait self-control (De Ridder et al., 2011). Participants responded to 13-items on 5-point Likert scales ranging from 1 (*strongly disagree*), to 5 (*strongly agree*) (e.g., "I am good at resisting temptation"). The scale has good internal reliability (De Ridder et al., 2011).

Attitudes against food waste

Participants completed a 5 item measure with a 7-point Likert response format (1 [*strongly disagree*], to 7 [*strongly agree*]) assessing attitudes against wasting food (e.g., "Even if I felt full, I would rather finish what is on my plate than see it go to waste".

Results

Study 1 normality judgments

Figure S1 displays the percentage of the sample judging each portion size as 'normal' across food types.

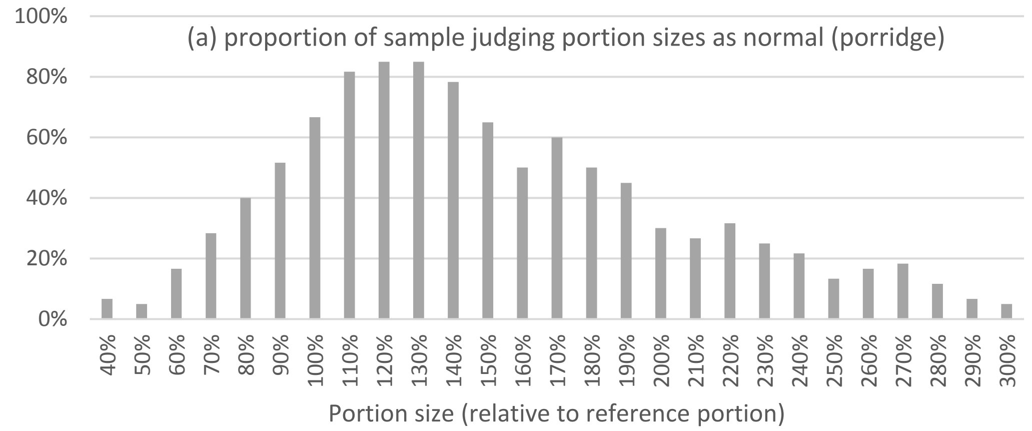
(a) proportion of sample judging portion sizes as normal (porridge)
100%
80%
60%
40%
20%
0%
40% 50% 60% 70% 80% 90% 100% 110% 120% 130% 140% 150% 160% 170% 180% 190% 200% 210% 220% 230% 240% 250% 260% 270% 280% 290% 300%
Portion size (relative to reference portion)

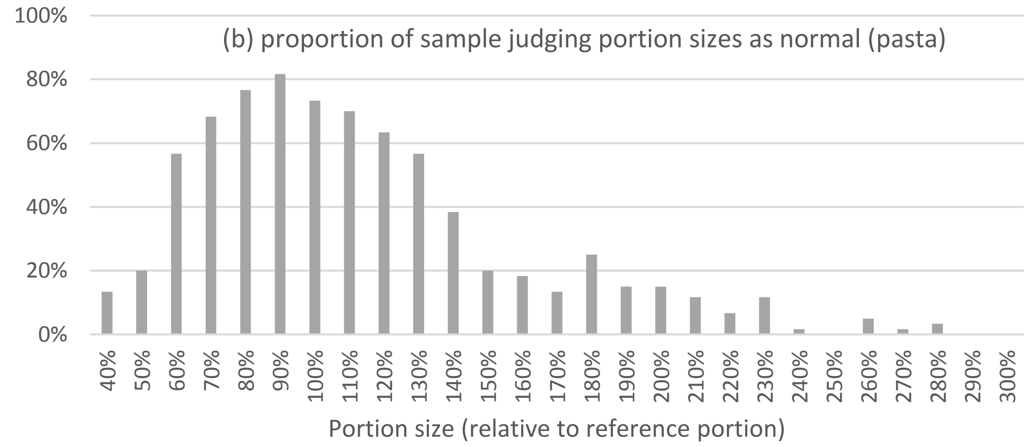
(b) proportion of sample judging portion sizes as normal (pasta)
100%
80%
60%
40%
20%
0%
40% 50% 60% 70% 80% 90% 100% 110% 120% 130% 140% 150% 160% 170% 180% 190% 200% 210% 220% 230% 240% 250% 260% 270% 280% 290% 300%
Portion size (relative to reference portion)

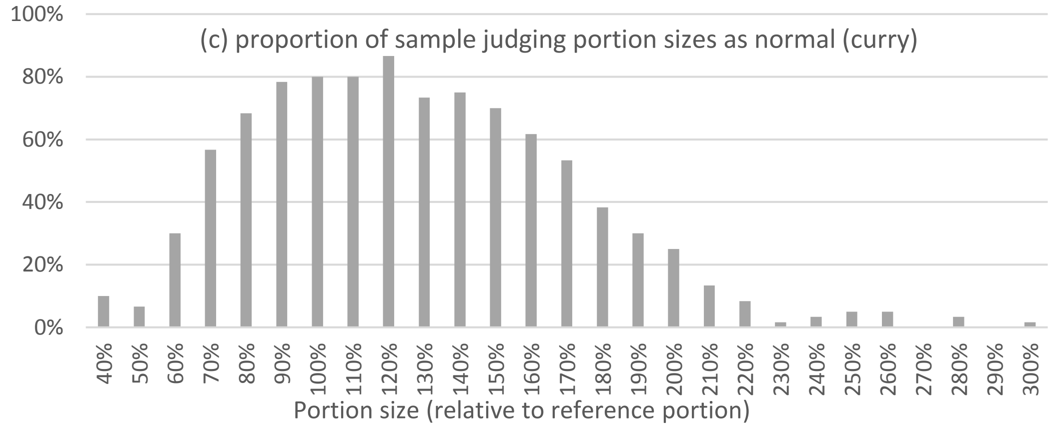
(c) proportion of sample judging portion sizes as normal (curry)
100%
80%
60%
40%
20%
0%
40% 50% 60% 70% 80% 90% 100% 110% 120% 130% 140% 150% 160% 170% 180% 190% 200% 210% 220% 230% 240% 250% 260% 270% 280% 290% 300%
Portion size (relative to reference portion)

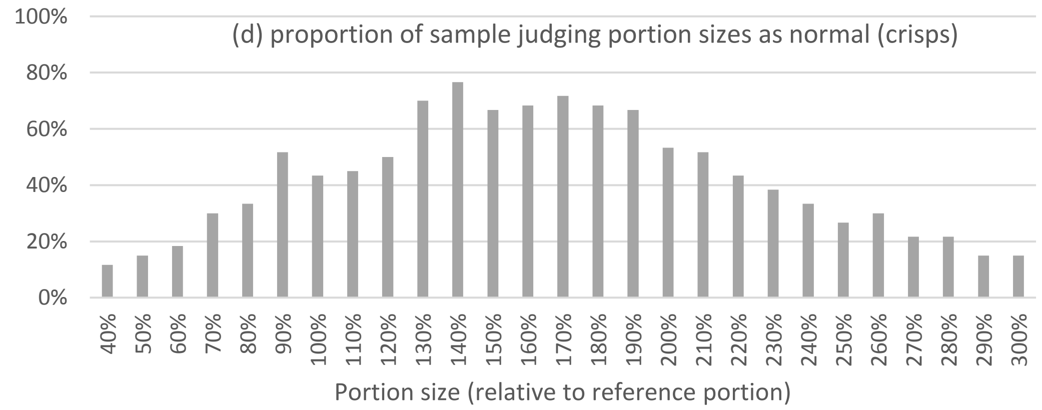
(d) proportion of sample judging portion sizes as normal (crisps)
100%
80%
60%
40%
20%
0%
40% 50% 60% 70% 80% 90% 100% 110% 120% 130% 140% 150% 160% 170% 180% 190% 200% 210% 220% 230% 240% 250% 260% 270% 280% 290% 300%
Portion size (relative to reference portion)

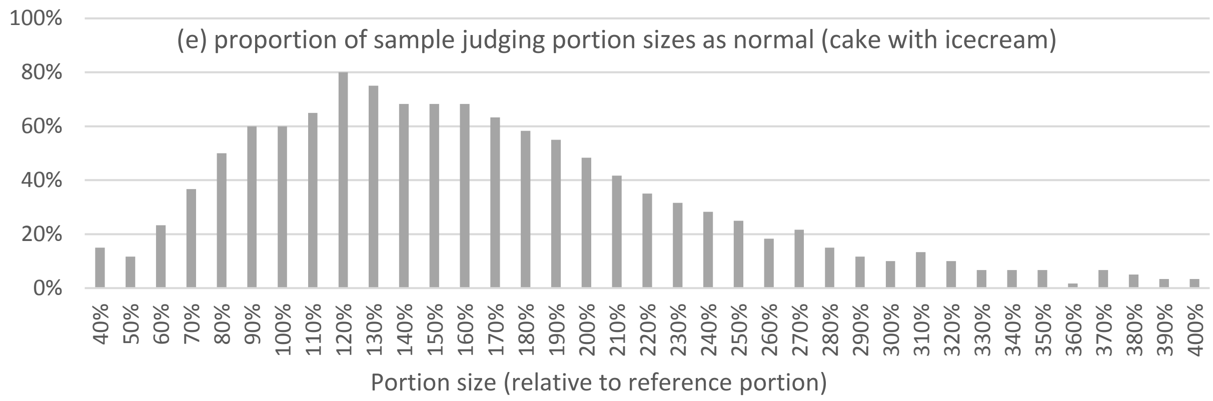

Figure S1. Percentage of participants in Study 1 judging each portion as ‘normal’ for 5 foods.

Study 2 analytic sample

One participant in Study 2 was missing intention data due to a technical fault, leaving an analytic sample size of 45 for the replication of Study 1 findings using collective norm ranges. The analytic sample size for the individual norm range analyses ranged from 29 to 37: four participants had missing reaction time data because they did not provide any correct relative size judgments within the time constraints for one or more portion size comparisons, and the remaining missing cases were excluded because participant ‘norm ranges’ included the minimum (40%) or maximum (300%) portion size stimulus, which meant that the participant was missing portion judgments that were categorised as ‘below’ or ‘above’ normal.

Replication of Study 1 findings in Study 2 using collective norm ranges

To determine the collective norm range (i.e., the range of portion sizes considered ‘normal’ by a majority of the sample) for Study 2, the lower boundary of the norm range for each food was marked by the smallest portion size falling within the norm range for at least 60% of participants, and the upper boundary as the largest portion size falling within the norm range for at least 60% of participants. In Study 2, the norm range for pasta was 90-140% (compared

to a range of 70 - 120% in Study 1), and for curry was 90-170% (compared to 80-160% in Study 1).

Consistent with Study 1, portion size category (‘below norm’, ‘within norm’, ‘above norm’) predicted intended consumption, $F(1.26, 55.63) = 886.19$, $p < .001$, $\eta_\rho^2 = .95$. Intended consumption did not differ between food types, $F(1,44) = 2.79$, $p = .10$, $\eta_\rho^2 = .06$, but there was a significant interaction between food and portion size category, $F(2, 88) = 13.34$, $p = <.001$, $\eta_\rho^2 = .23$. Follow up repeated-measures ANOVAs confirmed that portion size category predicted intended consumption for both pasta, $F(1.49, 65.65) = 716.12$, $p = <.001$, $\eta_\rho^2 = .94$, and curry, $F(1.29, 56.78) = 724.60$, $p = <.001$, $\eta_\rho^2 = .94$. For both food types, pairwise comparisons showed that intended consumption was significantly higher for portions that were ‘smaller than normal’ than for those considered ‘normal’, pasta: $MD = 1.40$, SE = 0.07, $p <.001$, curry: $MD = 1.71$, SE = 0.07, $p <.001$, and significantly higher for portions that were considered ‘normal’ than for those that were considered ‘larger than normal’, pasta: $MD = 1.67$, SE = 0.07, $p < .001$, curry: $MD = 1.71$, SE = 0.08, $p <.001$.

Boundary category based on the collective norm range predicted differences in intended consumption between pairs of portion sizes, $F(2, 88) = 5.70$, $p =.01$, $\eta_\rho^2 = .12$. Pairwise comparisons revealed the mean difference in intended consumption was larger between portion sizes that crossed the lower norm boundary than between portion size pairs that fell inside of the norm range, $MD = 0.19$, SE = 0.09, $p = .047$, which was not statistically significant against Bonferroni-adjusted $\alpha = .025$. However, unlike in Study 1, and in the main analyses reported in Study 2, differences in intended consumption were significantly larger between portion sizes that crossed the upper norm boundary than those that fell within the norm range boundary, $MD = 0.31$, SE = 0.08, $p <.001$. Type of food did not predict differences in intended consumption between portion size pairs, $F(1,44) = 0.41$, $p = .525$, $\eta_\rho^2 = .01$, and there was no significant interaction between food and boundary category, F(1.73,

76.26) = 1.19, p = .309, η_p^2 = .03. Neither cubic nor quadratic components were significant in any analysis, suggesting that a combination of linear and quartic functions best describe the pattern of discrimination performance observed across the 5 categories.

Additional post-hoc analysis: effect of norm range on discrimination performance polynomial trend analysis

Weber's law holds that as the size of a physical stimulus increases or decreases, discrimination performance diminishes or improves (Kingdom & Prins, 2016). Therefore, differences between smaller portion sizes should be easier to discriminate than differences between larger portion sizes. Because comparison position in the relative size judgment task (across lower boundary, within norm range, across upper boundary) is confounded with portion size, effects on discrimination performance could also be attributable to Weber's law and not only categorical perception.

To account for this possibility, we conducted additional post-hoc analyses to examine discrimination performance in 5 comparison positions: (a) below the norm range, (b) across the lower boundary, (c) within the norm range, (d) across the upper boundary, and (e) above the norm range. We conducted separate 5 (comparison position) $\times$ 2 (food type) repeated measures ANOVAs on relative size judgment RT and accuracy (adjusted alpha = .0125). Categorical perception would be evidenced by better discrimination performance for cross-boundary comparisons (b) and (d), than within-category comparisons (a), (c), and (e).

We also conducted trend analysis, which tests the overall pattern of the relationship between comparison position ([a] to [e]) and discrimination performance. For our 5-level independent variable (comparison category), it is possible for the pattern of discrimination performance across levels to be described by up to 4 different trend components: linear (a straight line, such that performance increases or decreases steadily from the smallest to the

largest comparison category), quadratic (a line with 1 change in direction), cubic (a line with 2 changes in direction), or quartic (a line with 3 changes in direction). For each trend component, we examined the associated *F* test for the polynomial contrast representing that trend. A statistically significant *F* test indicated that the pattern of discrimination performance across comparison positions conformed to that trend. A schematic of the trend components that we expected to describe the relationship between discrimination performance and comparison position is shown in Figure S2. In line with Weber’s law, we expected a significant linear trend, such that discrimination performance would diminish as portion size increased. In line with the prediction that portion size normality would be categorically perceived, we also expected a significant quartic trend. A quartic trend describes a ‘double peaked’ pattern, whereby there are 3 changes in direction in the line describing the pattern of performance across sequential levels. In this case, we expected to observe an upward trend or ‘peak’ in discrimination performance at comparison positions (b) and (d), corresponding to the boundaries of the norm range.

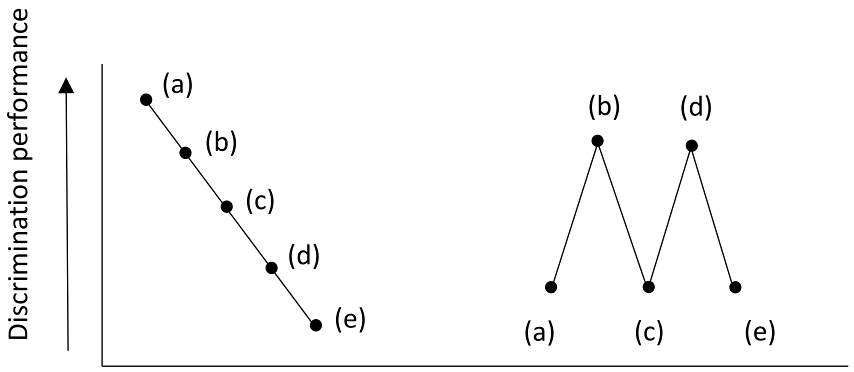

Figure S2. Schematic of linear (left) and quartic (right) trends across 5 sequential comparison positions: (a) below the norm range, (b) across the lower boundary, (c) within the norm range, (d) across the upper boundary, (e) above the norm range. Linear trend: no change in direction, quartic trend: 3 changes in direction across levels.

Post-hoc analysis results

In line with the primary results reported in the manuscript, there was a significant effect of norm range position on both relative size judgment accuracy, $F(2.56, 66.58) = 38.26$, $p < .001$, $\eta_\rho^2 = .60$, and RT, $F(1, 24) = 43.48$, $p < .001$, $\eta_\rho^2 = 64$, and a significant category × food interaction for RT. Results of pairwise comparisons are displayed in Figures S3 (accuracy, collapsed across foods) and S4 (RT plotted for curry and pasta seperately). The norm range boundaries significantly facilitated discrimination performance relative to within-category performance in ‘across lower boundary’ vs. ‘within norm range’ comparisons (for both accuracy and RT in both foods), and ‘across upper boundary’ vs. ‘above norm range’ comparisons (marginally significant for accuracy, and only significant for curry RT). These results are in line with the prediction that portion size normality is perceived categorically, but are also consistent with Weber’s law. However, inconsistent with both accounts, discrimination performance was not significantly different across the upper norm range boundary than ‘within the norm range’ (or ‘above the norm range’ for pasta RT). Consistent with Weber’s law (but not categorical perception), relative size judgment accuracy was marginally significantly better for portions in the ‘below norm’ category than those crossing the lower norm range boundary.

There was a significant linear trend across sequential portion size categories, such that discrimination performance diminished with increasing portion size (accuracy: $F(1, 26) = 124.80$, $p < .001$, $\eta_\rho^2 = .83$, RT: $F(1, 24) = 43.48$, $p < .001$, $\eta_\rho^2 = .64$. The linear trend in RT was significant for both curry, $F(1, 29) = 83.39$, $p < .001$, $\eta_\rho^2 = .74$, and pasta, $F(1, 28) = 23.31$, $p < .001$, $\eta_\rho^2 = .45$, consistent with Weber’s law. Consistent with the prediction that portion size normality is perceived categorically, there was a significant quartic trend, such that the tendency for discrimination performance to worsen with increasing portion size is not purely linear across all ascending portion size categories, but shows a slight change in

direction (rather than continuing linearly) at the categorical boundaries (b) and (d). The quartic trend was significant for both accuracy, $F(1, 26) = 18.77$, $p < .001$, $\eta_\rho^2 = .42$, and RT, $F(1, 24) = 9.24$, $p = .01$, $\eta_\rho^2 = .28$ (although this was only significant for curry RT, $F(1, 29) = 17.26$, $p < .001$, $\eta_\rho^2 = .37$, not pasta RT, $F(1, 28) = 0.30$, $p = .59$, $\eta_\rho^2 = .01$). No other trend component (cubic, quadratic) was significant. Trend analysis therefore indicates that while the pattern of discrimination performance across the 5 categories is strongly linear, there is also a significant non-linear quartic component, consistent with categorical perception of portion size normality.

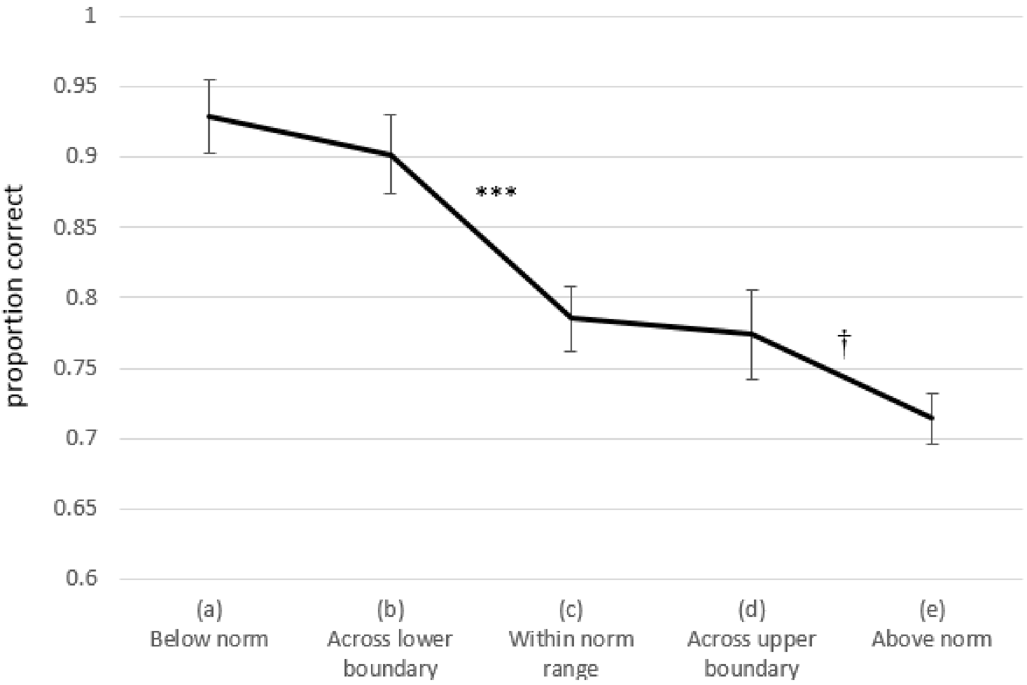

Figure S3. Relative size judgment accuracy by norm boundary position collapsed across foods. For comparisons between adjacent norm boundary categories: *** $p < .001$, † $p < .03$. Error bars represent standard error of the mean.

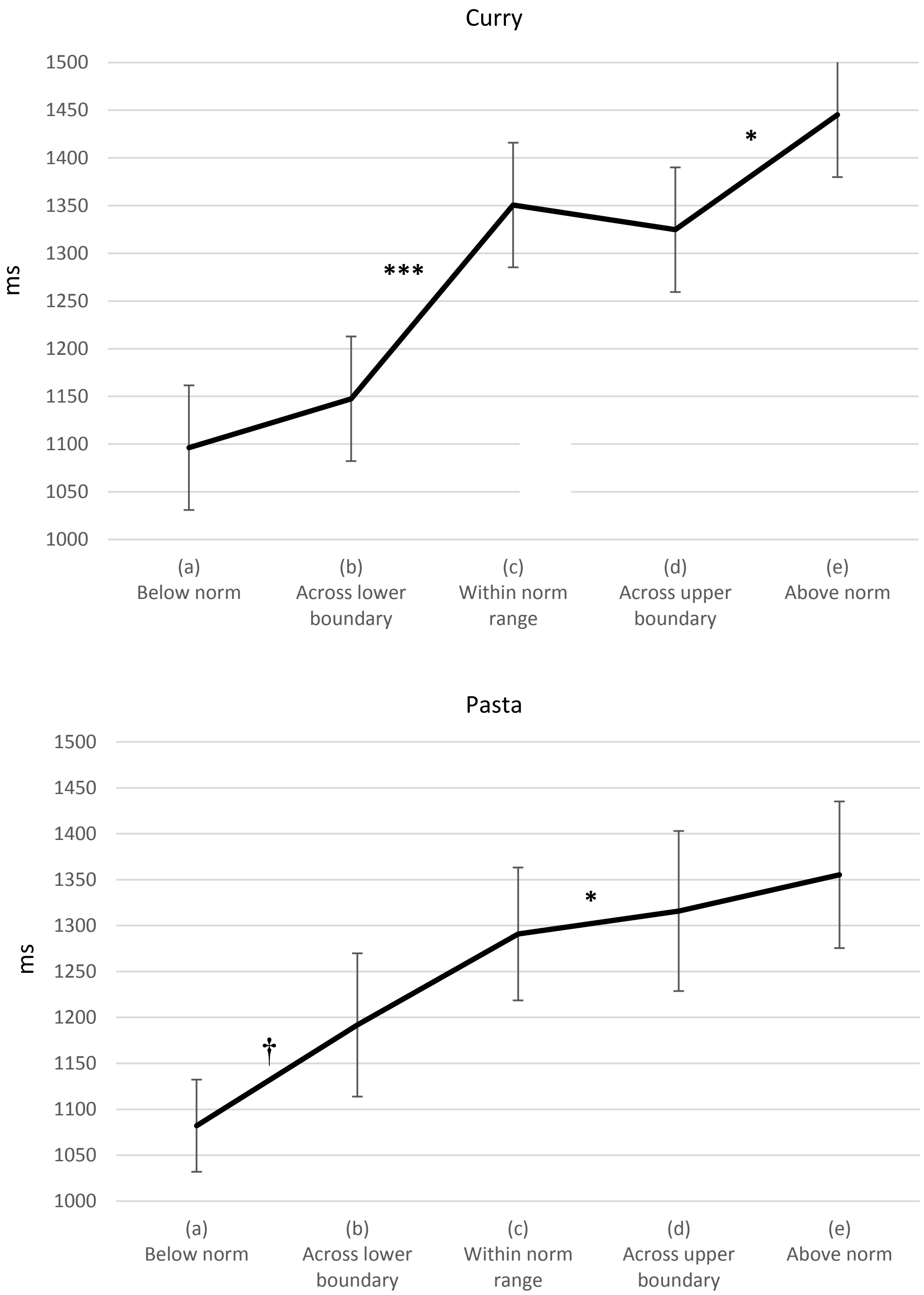

Figure S4. Relative size judgment RT by norm boundary position and food type (curry [top], pasta [bottom]). For comparisons between adjacent norm boundary categories: *** p <.001, * significant difference at p <.0125, † p <.03. Error bars represent standard error of the mean.

Table S2.
Complete ANOVA results.

Dependent variable	Main effect: portion size category		Main effect: food		Interaction food × portion size category	
	F (df1, df2)	η_ρ^2	*F* (df1, df2)	η_ρ^2	*F* (df1, df2)	η_ρ^2
Study 1						
Intended consumption	1137.40 (1.31, 77.25)	.95***	0.62 (2.89, 170.62)	.01	29.41 (5.94, 350.34)	.33***
Diff. intended consumption	11.32 (2, 118)	.16***	5.41 (4, 236)	.08*** [a]	1.52 (8, 472)	.03
Study 2						
Intended consumption	525.08 (1.56, 45.26)	.95***	2.72 (1, 29)	.09	3.21 (2, 58)	.10*
Diff. intended consumption	4.20 (1.50, 49.33)	.11*	4.95 (1, 33)	.13* [b]	2.88 (2, 66)	.08
Relative size judgment accuracy	24.48 (1.31, 43.32)	.43***	0.96 (1,33)	.03	0.53 (1.53, 50.58)	.02
Relative size judgment RT	16.09 (1.39, 44.32)	.34***	2.66 (1, 32)	.08	6.45 (2, 64)	.17*

****p* <.001, **p* <.05.

[a] intended consumption was more sensitive to changes in portion size of pasta than crisps, *MD* = 0.27, SE = 0.06, *p* < .001, and [b] of curry than of pasta, *MD* = 0.16, SE = 0.07, *p* = .03.
